# Infection complicating locked intramedullary nailing of open lower-extremity fractures: incidence, associated risk factors, and lessons for improving outcome in a low-resource setting 

**DOI:** 10.5194/jbji-8-71-2023

**Published:** 2023-02-14

**Authors:** Stephen A. Adesina, Isaac O. Amole, Akinsola I. Akinwumi, Adepeju O. Adegoke, James I. Owolabi, Imri G. Adefokun, Adewumi O. Durodola, Olufemi T. Awotunde, Samuel U. Eyesan

**Affiliations:** 1 Department of Family Medicine, Bowen University, Iwo, 232102, Nigeria; 2 Department of Family Medicine, Afe Babalola University, Ado-Ekiti, 360101, Nigeria; 3 Department of Surgery, Bowen University, Iwo, 232102, Nigeria; 4 Department of Surgery, Bowen University Teaching Hospital, Ogbomoso, 210001, Nigeria

## Abstract

**Introduction**: Infection is the chief complication that makes open fractures difficult to treat. Most low- and middle-income countries (LMICs) are missing out on modern management techniques developed to achieve better outcomes in high-income countries (HICs). One of these is the use of locked intramedullary (IM) nails. This study aimed to determine the factors associated with infection of open fractures treated with the surgical implant generation network (SIGN) nail at a Nigerian tertiary hospital. **Methods**: Data were collected prospectively on 101 open fractures of the femur and tibia over an 8-year period. Active surveillance for infection was done on each patient. Infection was diagnosed as the presence of wound breakdown or purulent discharge from (or near) the wound or surgical incision. Potential risk factors were tested for association with infection. **Results**: There were 101 fractures in 94 patients with a mean age of 37.76 years. The following treatment-related factors demonstrated significant associations with infection – timings of antibiotic administration ( p<0.001 ) and definitive fracture fixation ( p=0.002 ); definitive wound closure ( p<0.001 ), fracture-reduction methods ( p=0.005 ), and surgery duration ( p=0.007 ). **Conclusions**: Although this study has limitations precluding the drawing up of final conclusions, the findings suggest that the risk factors for infection of nailed open fractures in LMICs are similar to those in HICs. Consequently, outcomes can potentially improve if LMICs adopt the management principles used in HICs in scientifically sound ways that are affordable and socially acceptable to their people. Further studies are suggested to establish our findings.

## 1 Introduction

The occurrence of long bone fractures is increasing in low- and middle-income countries (LMICs) due to rampant high-energy trauma resulting mainly from road traffic accidents (Ibeanusi and Obalum, 2019; Ifesanya and Alonge, 2012; Agarwal-Harding et al., 2016; Makridis et al., 2013). The high energy damages both the bone and soft-tissue envelope, with consequent open fractures in many cases (Makridis et al., 2013). An estimated 80 % of severe fractures occur in the developing world (Gellman, 2016). The soft-tissue damage and contamination of the fracture site raise the likelihood of complications, chief among which is infection (Kakar and Tornetta, 2007; Zalavras and Patzakis, 2003). Even with the modern soft-tissue management techniques and modern implants available in high-income countries (HICs), achieving good outcomes for open fractures can be particularly difficult (Melvin et al., 2010). Thus, treating open fractures in the austere settings of LMICs can be tough and outcomes have been noted to be particularly bad in these countries (Zirkle, 2008).

Notwithstanding the advances in infection control practices in HICs, surgical site infections (SSIs) remain a significant cause of morbidity, prolonged hospitalization, and death (Centers for Disease Control and Prevention, 2017). With infection being such a frightening complication, hospitals in HICs implement surveillance protocols in order to improve infection prevention practices (Brandt et al., 2006). This is because feedback of appropriate data to surgeons from SSI surveillance is a proven component of the strategies to reduce SSI risk. The Centers for Disease Control and Prevention (CDC) emphasizes active, patient-based, prospective SSI surveillance for superficial incisional, deep incisional, and organ/space SSI events. Duration of surveillance for SSIs was reduced from the previously recommended 1 year to 90 d in the updated CDC/National Healthcare Safety Network (NHSN) definitions for SSIs (Centers for Disease Control and Prevention, 2017).

Due to its biomechanical and biological advantages, locked intramedullary (IM) nailing is now the gold standard definitive treatment for most open fractures of long bones in HICs, preferred to plate or external fixation (Makridis et al., 2013; Kakar and Tornetta, 2007). Compared to external fixation, it does not demand the same high level of patient compliance, it obviates the need for multiple surgeries and is aesthetically more acceptable to the patients (Zalavras and Patzakis, 2003). Moreover, IM nailing is associated with less chance of infection and shorter healing time than external fixation (Tornetta et al., 1994). Increased incidence of infection and implant failure have been the discouraging events with plate fixation (Bach and Hansen, 1989; Clifford et al., 1988). Unfortunately, locked IM nailing is used sparingly for open fractures by surgeons in LMICs owing to the risk of infection and/or unavailability (Ikem et al., 2007; Salawu et al., 2017; Ikpeme et al., 2011).

Nevertheless, some surgeons in LMICs who have access to locked IM nails have begun using them conscientiously on open fractures, with outcomes that are within acceptable limits. In a prospective cohort study involving multiple hospitals in LMICs worldwide, Whiting et al. (2019) found an overall infection rate of 11.9 % after fixation of 1061 open tibia shaft fractures with a locked IM nail. Consequently, despite the resource constraints in many LMICs, locked IM nailing, if available, is a viable approach to fixing most open fractures (Whiting et al., 2019; Young et al., 2013). This study aimed to determine the incidence of infection and associated risk factors from a 90 d prospective surveillance of open fractures treated with the surgical implant generation network (SIGN) nail at a Nigerian tertiary hospital. The SIGN nail is a solid locked reamed IM nail manufactured and freely distributed by SIGN Fracture Care International (Richland, WA, USA).

## 2 Methods

### 2.1 Study center

The study center was a teaching hospital in southwestern Nigeria. It served the people of a semi-urban city and nearby villages/towns that were home to subsistence farmers, small business owners, civil servants, and artisans.

### 2.2 Management protocols

The patients were started on broad-spectrum antibiotics on arrival at our emergency room and had their fractured lower limbs splinted. After the initial resuscitation, thorough wound debridement and irrigation were done in the operating room (OR). If the patient had presented ≤8 h post injury and had no skin loss, immediate primary wound closure was done. Otherwise, a delayed primary closure or closure with muscle flap and split-thickness skin graft was done at the time of definitive fracture fixation. Depending on the severity of their injuries, patients had either further OR debridement or daily wound dressing changes by nurses in the wards. Definitive fracture fixations were done with the SIGN nail any time from day 0 post injury using the surgical procedure described by the manufacturer (Feibel and Zirkle, 2009; Zirkle and Shearer, 2009; SIGN Fracture Care International, 2016). When adjudged correct, the primarily closed wounds were not re-opened; rather, closed reduction was done. Otherwise, open reduction was done. Postoperatively, the patients were continued on intravenous broad-spectrum antibiotics for 5–7 d and subsequently on oral antibiotics until their wounds healed.

Active in-patient surveillance for infection was done by monitoring for signs of infection. Further clinical examination was done on patients with suggestive sign(s) to detect wound breakdown or purulent discharge from or near the wound or incision. The patients were discharged from the hospital starting from postoperative day 5. The importance of returning for a follow-up consultation was explicitly communicated to the patients and their relations. They were actively encouraged to attend even if they felt all was well with their injured limb. At the follow-up, each patient was reassessed for infection. Follow-ups were done for at least 3 months, usually at 4–6 weekly intervals. If infection occurred, additional follow-ups were scheduled based on the healing progress. All the patients were also instructed to return if they had discharge from their operated limb. Infection was diagnosed clinically as the presence of wound breakdown or purulent discharge from (or near) the wound or surgical incision.

### 2.3 Study design

This was a prospective observational study. The inclusion criteria were open fractures of the femur and tibia treated with the SIGN nail between July 2014 and June 2022 (8 years). The exclusion criteria included open fractures treated by means other than SIGN nailing, open fractures whose skin wounds had healed before presenting to us, and fractures in patients who could not be followed up due to early postoperative death. Data were collected on potential risk factors and were tested for association with infection. The risk factors included (i) patient- and injury-related factors (age, sex, fracture aetiology, comorbidity, concomitant injury, time of injury, fractured bone, fracture severity) and (ii) treatment-related factors (time when antibiotics were first administered, method of wound closure, time when definitive fracture fixation was undertaken, duration of surgery, fracture-reduction method, combination of side plate with SIGN nail).

Fracture severity was classified according to the modified Gustilo–Anderson system (Zalavras and Patzakis, 2003; Diwan et al., 2018; Gustilo et al., 1984): type I – puncture wounds ≤1 cm, with minimal contamination and muscle damage; type II – lacerations >1 with moderate soft-tissue injury, adequate bone coverage and minimal comminution; type IIIA – extensive soft-tissue damage from high-velocity injury with severe crushing component, heavily contaminated wounds, severe comminution/segmental fractures but with adequate bone coverage. We included gunshot fractures here. We presumed fractures that had been open for ≥8 h prior to the commencement of treatment were heavily contaminated and classified them as type IIIA. Type IIIB refers to extensive soft-tissue damage, with stripping of the periosteum and exposure of the bone, usually associated with heavy contamination and severe comminution of the bone as well as inadequate soft-tissue cover; and type IIIC indicates any open fracture with arterial injury requiring repair, regardless of the degree of soft-tissue injury.

The time length between the occurrence of the fracture and the administration of antibiotics (fracture-to-antibiotics interval) was categorized as follows: ≤3 h, >3 but ≤6 h, and >6 h. The method of wound closure was categorized as immediate primary, delayed primary, and flap/skin graft. The time length between the occurrence of the fracture and the definitive fracture fixation (fracture-to-fixation interval) was categorized into 0–2, 3–7, and >7 d. The time length between skin incision and closure during definitive fixation (*duration of surgery*) was categorized into ≤1 h, >1 h but ≤2 h, and >2 h. Based on the reduction method, the fractures were divided into closed- and open-reduction categories.

### 2.4 Statistical analysis

The data were analyzed with SPSS version 23 (IBM Corp, New York, USA). When the last patient had completed the follow-up, incidence of infection was calculated as the percentage of the number of fractures in which infection occurred (numerator) and the total number of open fractures fixed with the SIGN nail over the study period (denominator). The data on potential risk factors and infection were subjected to cross-tabulations and Pearson's chi square ( $\chi^{2}$ ) inferential statistics (or Fisher's exact test when the sample was small) to determine which factors demonstrated a statistically significant association with infection. All p values were two-tailed and the level of significance was set at p<0.05 .

## 3 Results

Over the 8-year study period, a total of 102 open fractures of the femur and tibia in 95 patients were treated with the SIGN nail. This was made up of 43 (42.2 %) femur and 59 (57.8 %) tibia fractures. However, the analysis included 101 fractures in 94 patients: one type IIIA tibia fracture in a 46-year-old man who died 6 d postoperatively was excluded. (He died of suspected pulmonary thromboembolism, although his relations declined autopsy.) Eighty-eight fractures healed without infection while 13 fractures got infected, making the incidence of infection 12.9 % of the fractures. However, the relatively small sample size and descriptive single-center nature of our study are limitations that precludes drawing up of final conclusions. Further studies – preferably large, randomized trials – are needed to conclusively ascertain our findings.

The analysis displayed in Table 1 shows that the age of the patients ranged from 14 to 76 years with a mean of 37.76 years, and that highest percentage (33.3 %) of infection occurred among the 50–59-year age group. Infection was more present in males (14.1 %) than in females (8.7 %) and in those injured during the evening/night (13.2 %) than those injured in the morning/noon (12.2 %) times. The percentages of infection were higher among those injured in motor vehicle (16.7 %) and pedestrian accidents (15.4 %) than other causes. The patients who sustained other fractures/dislocations also got infected (31.3 %) significantly ( p=0.035 ) more than those with other concomitant injuries. None of the other associations between patient- or injury-related factors with infection was statistically significant.

**Table 1:** Cross-tabulation of patient- or injury-related factors and infection ( N=94 ).

Variables		No infection	Infection present	Test statistic	p value
		n (%)	n (%)	( $\chi^{2}$ )	
Age groups (years)	10–19	6 (100.0)	0 (0.0)		
Mean age:	20–29	20 (100.0)	0 (0.0)		
37.76 years	30–39	23 (79.3)	6 (20.7)	^**^	0.102
Age range:	40–49	21 (91.3)	2 (8.7)		
14–76 years	50–59	6 (66.7)	3 (33.3)		
	60–69	4 (80.0)	1 (20.0)		
	70–79	2 (100.0)	0 (0.0)		
Gender	Male	61 (85.9)	10 (14.1)	0. 453	0.501
	Female	21 (91.3)	2 (8.7)		
Time of injury	Morning/noon	36 (87.8)	5 (12.2)	0. 021	0.884
	Evening/night	46 (86.8)	7 (13.2)		
Cause of fracture	Gunshot	1 (100.0)	0 (0.0)		
	Fall	1 (100.0)	0 (0.0)	^**^	0.789
	Motor vehicle accident	15 (83.3)	3 (16.7)		
	Motorcycle accident	54 (88.5)	7 (11.5)		
	Pedestrian accident	11 (84.6)	2 (15.4)		
Concomitant injury	None	50 (94.3)	3 (5.7)		
	Head	7 (87.5)	1 (12.5)	^**^	**0.035**
	Soft tissue	14 (82.4)	3 (17.6)		
	Other fractures/dislocations	11 (68.8)	5 (31.3)		
Comorbidity	No comorbidity/controlled hypertension	73 (85.9)	12 (14.1)	1.457	0.227
	Other comorbidities	9 (100.0)	0 (0.0)		

$\chi^{2}=$ Pearson chi-square. ^**^ Fisher's exact test. Statistically significant p values are indicated in bold.

**Figure 1:**
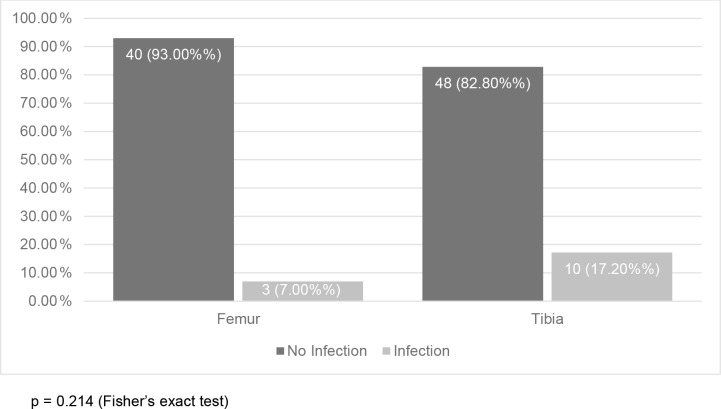
Association between fracture bone nailed and infection ( N=101 ). p=0.214 (Fisher's exact test).

**Figure 2:**
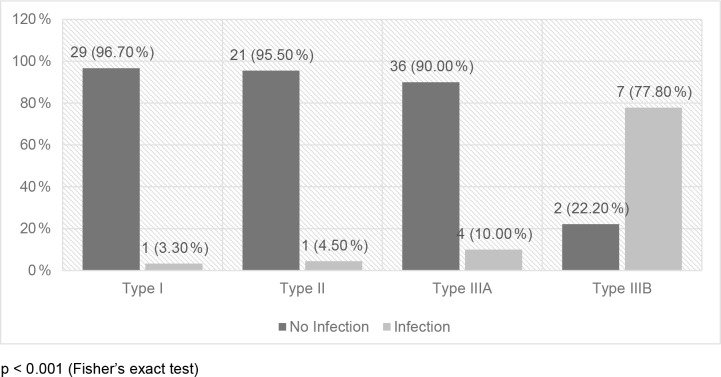
Association between fracture severity and infection ( N=101 ). p<0.001 (Fisher's exact test).

Three out of 43 femur fractures and 10 out of 58 tibia fractures became infected, but this association was not statistically significant (Fig. 1). On the other hand, fracture severity had a statistically significant association ( p<0.001 ) with infection. The infected cases were 3.3 %, 4.5 %, 10.0 %, and 77.8 % among Gustilo–Anderson types I, II, IIIA, and IIIB, respectively (Fig. 2).

Virtually all the treatment-related factors demonstrated statistically significant associations with infection (Table 2). The percentage of infection increased with increases in fracture-to-antibiotics interval ( p<0.001 ) and duration of surgery ( p=0.007 ). Fewer fractures (3.7 %) in the “immediate primary” closure category became infected ( p<0.001 ) than in the “delayed primary ” closure (30.3 %) or “flap/skin graft” coverage (77.8 %) categories. A significantly higher percentage (31.8 %) of fractures fixed beyond 7 d of injury, compared to those fixed earlier, became infected ( p=0.002 ). While infection complicated only two (3.8 %) fractures in the closed-reduction group, 11 (22.4 %) of those in the open-reduction category were infected ( p=0.005 ). Although more (18.2 %) of the fractures in which a side plate was used than those without plates became infected, the association was not statistically significant ( p=0.577 ).

Table 3 shows the details of the 13 fractures in which infection occurred. They were treated by nail removal and oral antibiotics. The infection subsequently resolved in all of them.

**Table 2:** Cross-tabulation of treatment-related factors and infection ( N=101 ).

Variables ( N=101 )		No infection	Infection present	Test statistic	df	p -value
		n (%)	n (%)	( $\chi^{2}$ )		
Fracture-to-antibiotics interval	≤3 h	51 (98.1)	1 (1.9)	17.919	2	**0.000**
	>3 but ≤6 h	27 (84.4)	5 (15.6)			
	>6 h	10 (58.8)	7 (41.2)			
Method of wound closure	Immediate primary	79 (96.3)	3 (3.7)	^**^	^**^	**0.000**
	Delayed primary	7 (70.0)	3 (30.0)			
	Flap/skin graft	2 (22.2)	7 (77.8)			
Fracture-to-fixation interval	0–2 d	26 (83.9)	5 (16.1)	12.317	2	**0.002**
	3–7 d	47 (97.9)	1 (2.1)			
	>7 d	15 (68.2)	7 (31.8)			
Fracture-reduction method	Closed	50 (96.2)	2 (3.8)	7.785	1	**0.005**
	Open	38 (77.6)	11 (22.4)			
Duration of surgery	≤1 h	45 (95.7)	2 (4.3)	10.036	2	**0.007**
	>1 but ≤2 h	37 (84.1)	7 (15.9)			
	>2 h	6 (60.0)	4 (40.0)			
Side plate used?	No	79 (87.8)	11 (12.2)	0. 310	1	0.577
	Yes	9 (81.8)	2 (18.2)			

$\chi^{2}=$ Pearson chi-square. ^**^ Fisher's exact test. Statistically significant p values are indicated in **bold**.

**Table 3:** Follow-up of infected cases ( n=13 ).

Patients	Age	Sex	Bone	Type	Follow-up time at when	Mode of	End point of
					infection was diagnosed	treatment	treatment
					(days post op)		
1.	32	M	Tibia	IIIB	475	A + B	Infection resolved
2.	31	M	Tibia	IIIB	90	A + B	Infection resolved
3.	31	M	Tibia	IIIB	90	A + B	Infection resolved
4.	34	M	Tibia	IIIB	36	A + B	Infection resolved
5.	50	M	Tibia	IIIB	33	A + B	Infection resolved
6.	48	M	Femur	I	45	A + B	Infection resolved
7.	54	F	Tibia	IIIB	74	A	Infection resolved
8.	41	M	Tibia	IIIB	57	A + B	Infection resolved
9.	52	M	Tibia	IIIA	133	A + B	Infection resolved
10.	61	F	Femur	IIIA	85	A	Infection resolved
11.	35	M	Tibia	II	86	A	Infection resolved
12.	21	M	Tibia	IIIA	88	A + B	Infection resolved
13.	36	M	Femur	IIIA	85	A + B	Infection resolved

A: oral antibiotics; B: nail removal, reaming and irrigation.

## 4 Discussion

Except for centers that are beneficiaries of free donation of orthopedic implants, locked IM nails are still scarce goods in many LMICs. They are reserved for a few rich patients who could afford to pay out of pocket for them (Zirkle, 2008). When available, many surgeons in these countries use them mostly to treat closed fractures and few carefully selected open fractures, owing to the risk of infection (Ikem et al., 2007; Salawu et al., 2017; Ikpeme et al., 2011; Ibeanusi, 2018). The present study included only open fractures fixed with locked IM nail. In our series, the incidence of infection was 12.9 %, and fracture severity was observed to have a statistically significant association ( p<0.001 ) with infection. The infection rates were 3.3 %, 4.5 %, 10.0 %, and 77.8 %, respectively among Gustilo–Anderson types I, II, IIIA, and IIIB fractures. These figures compare favorably with what has been previously documented, except for type IIIB fractures (Zalavras and Patzakis, 2003; Whiting et al., 2019; Patzakis and Wilkins, 1989; Haonga et al., 2020; Seron and Rasool, 2018). Earlier locked nail studies in Nigeria lumped closed and open fractures together without stating the specific infection rate among the open fractures (Ikem et al., 2007; Ikpeme et al., 2011; Ibeanusi, 2018).

The infection incidence in our study was particularly inflated by the relatively small sample size and high rate among type IIIB fractures. Owing to their severity, type IIIB fractures are known to be particularly difficult to treat and are associated with high infection rate (Zalavras and Patzakis, 2003; Whiting et al., 2019; Patzakis and Wilkins, 1989). Authors of a recent systematic review on the management of Gustilo–Anderson IIIB open tibial fractures in adults concluded that the standards of care should center on early antibiotic prophylaxis, consultant-led orthoplastic input with early debridement, fracture fixation, and soft-tissue coverage within 72 h of injury (Myatt et al., 2021). Before open fractures can be fixed with IM nail, definitive soft-tissue coverage must be guaranteed as this is the most important factor in reducing subsequent deep infection (Yokoyama et al., 2006).

In our austere setting though, the late arrival of patients to the hospital and inadequate human/material resources constrained such early definitive closure for injuries requiring flap coverage. For this reason, early definitive bony stabilization, which is also known to reduce infection (Whiting et al., 2019), was not possible for most of the type IIIB fractures. The two uninfected cases had definitive coverage and IM nailing within the first week of injury. For types IIIA or lower, we would usually do immediate primary wound closure which has been documented by many recent studies to reduce infection rate (Whiting et al., 2019; Jenkinson et al., 2014; Scharfenberger et al., 2017).

The definitive optimal approach to the treatment of type IIIB fractures is an ongoing area of clinical research and deep infection remains the biggest concern, notwithstanding the treatment modality (Myatt et al., 2021). External fixators could have been used to treat the type IIIB fractures, but they were not readily available. They also were neither socially acceptable nor economically affordable to our patients owing to their need for prolonged hospital stay and multiple surgeries. The SIGN nails on the other hand were used for patients free of charge and it achieved earlier functional restoration of the injured limbs as well as an earlier return to work. Thus, the patients preferred an infected but usable limb afforded by nailing to a sterile non-union from protracted external fixation. However, the infected fractures healed and the infection resolved following nail removal and antibiotic treatment.

Except for the use of a side plate, every other treatment-related factor evaluated in our series demonstrated a statistically significant association with infection. These included timing of antibiotic administration ( p<0.001 ), method of definitive wound closure ( p<0.001 ), timing of definitive fracture fixation ( p=0.002 ), duration of surgery ( p=0.007 ), and fracture-reduction method ( p=0.005 ). The soft-tissue damage and accompanying introduction of environmental contaminants into open injuries produces an increased risk of infection. Hence, early antibiotic administration reduces the risk of infection in open fractures (Patzakis and Wilkins, 1989; Chang et al., 2019; Garner et al., 2020). In our series, whereas only 1 out of 52 (1.9 %) patients became infected after the administration of antibiotics within 3 h of injury, the figures were 7 out of 17 (41.2 %) for those that had antibiotics after 6 h.

Furthermore, we found that a significantly smaller percentage (3.7 %) of wounds that had immediate primary closure were infected compared to those that had delayed primary closure (30.0 %) or flap coverage (77.8 %). Some recent studies have supported immediate primary wound closure (of appropriately selected cases) as an acceptable strategy to reduce deep infection risk (Whiting et al., 2019; Jenkinson et al., 2014; Scharfenberger et al., 2017). Scharfenberger et al. (2017) and Jenkinson et al. (2014) opined that immediate wound closure may protect against nosocomial infections. Also, Diwan et al. (2018) supported this opinion by asserting that infection mostly develops due to hospital-acquired organisms in the developed world and due to reduced (or delayed) access to modern care in the developing world. Similarly, many studies have shown that SSIs are minimized by early definitive fracture stabilization (Zalavras and Patzakis, 2003; Whiting et al., 2019; Diwan et al., 2018), shorter operative time (Ravi et al., 2019; Cheng et al., 2017), and closed (or limited open) fracture reduction (Gellman, 2016; Adesina et al., 2021; Winquist et al., 1984).

## 5 Conclusions

The foregoing findings from our study suggest that the risk factors for infection of open fractures in LMICs are essentially the same as those found by earlier studies in HICs. Our findings also insinuate that it is possible for LMICs to achieve acceptably satisfactory outcomes for these fractures despite the scarce resources. This can happen if surgeons in LMICs learn to adopt the more sophisticated management approaches of HICs in ways that are affordably cheaper but scientifically sound, more easily accessible and socially acceptable to their peoples. For example, surgeons in LMICs can choose to do immediate primary wound closure following thorough debridement instead of insisting on delayed closure for which they often lack reliably efficient sterile wound dressing materials used in HICs. Furthermore, our finding of a high infection rate in type IIIB fractures advises improvement of the protocol for severe soft-tissue injury management or seeking alternative treatment modalities with a lower infection rate.

## Data Availability

Data are available upon request.
